# Large-scale characterization of the microvascular geometry in development and disease by tissue clearing and quantitative ultramicroscopy

**DOI:** 10.1177/0271678X20961854

**Published:** 2020-10-12

**Authors:** Artur Hahn, Julia Bode, Allen Alexander, Kianush Karimian-Jazi, Katharina Schregel, Daniel Schwarz, Alexander C Sommerkamp, Thomas Krüwel, Amir Abdollahi, Wolfgang Wick, Michael Platten, Martin Bendszus, Björn Tews, Felix T Kurz, Michael O Breckwoldt

**Affiliations:** 1 Neuroradiology Department, University Hospital Heidelberg, Heidelberg, Germany; 2 Department of Physics and Astronomy, University of Heidelberg, Heidelberg, Germany; 3 Schaller Research Group at the University of Heidelberg and the German Cancer Research Center (DKFZ), Molecular Mechanisms of Tumor Invasion, Heidelberg, Germany; 4 Clinical Cooperation Unit Neuroimmunology and Brain Tumor Immunology, German Cancer Research Center (DKFZ), Heidelberg, Germany; 5 Faculty of Biosciences, Heidelberg University, Heidelberg, Germany; 6 German Cancer Consortium and Heidelberg Institute of Radiation Oncology, National Center for Radiation Research in Oncology, Heidelberg, Germany; 7 Heidelberg University School of Medicine, Heidelberg University, Heidelberg, Germany; 8 Translational Radiation Oncology, German Cancer Research Center (DKFZ), Heidelberg, Germany; 9 Neurology Clinic and National Center for Tumor Diseases, University Hospital Heidelberg, Heidelberg, Germany; 10 Clinical Cooperation Unit Neurooncology, German Cancer Consortium (DKTK), German Cancer Research Center (DKFZ), Heidelberg, Germany; 11 Department of Neurology, University Medical Center Mannheim, Heidelberg University, Heidelberg, Germany

**Keywords:** Ultramicroscopy, microvascular networks, clearing, selective plane illumination microscopy, angiogenesis

## Abstract

Three-dimensional assessment of optically cleared, entire organs and organisms has recently become possible by tissue clearing and selective plane illumination microscopy (“ultramicroscopy”). Resulting datasets can be highly complex, encompass over a thousand images with millions of objects and data of several gigabytes per acquisition. This constitutes a major challenge for quantitative analysis. We have developed post-processing tools to quantify millions of microvessels and their distribution in three-dimensional datasets from ultramicroscopy and demonstrate the capabilities of our pipeline within entire mouse brains and embryos. Using our developed acquisition, segmentation, and analysis platform, we quantify physiological vascular networks in development and the healthy brain. We compare various geometric vessel parameters (e.g. vessel density, radius, tortuosity) in the embryonic spinal cord and brain as well as in different brain regions (basal ganglia, corpus callosum, cortex). White matter tract structures (corpus callosum, spinal cord) showed lower microvascular branch densities and longer vessel branch length compared to grey matter (cortex, basal ganglia). Furthermore, we assess tumor neoangiogenesis in a mouse glioma model to compare tumor core and tumor border. The developed methodology allows rapid quantification of three-dimensional datasets by semi-automated segmentation of fluorescently labeled objects with conventional computer hardware. Our approach can aid preclinical investigations and paves the way towards “quantitative ultramicroscopy”.

## Introduction

Vascular remodeling is a key feature of development and disease.^1^,^2^ Neoangiogenesis is a hallmark of cancer and its investigation, both qualitatively and quantitatively is crucial for preclinical and clinical cancer studies. Tumor neoangiogenesis is also closely linked to tumor progression and metastasis formation.^3^ Histological (two-dimensional (2D)) analysis of serially segmented sections and 3D image reconstruction is currently the gold standard to quantify vascular networks. However, this is both time consuming and labor intensive. Moreover, reconstructed volumes are generally small.^4^ Current 3D-imaging techniques such as magnetic resonance imaging (MRI) and positron emission tomography (PET) do not possess enough spatial resolution to visualize the small capillaries of the microvasculature, though perfusion techniques enable functional probing of vascular parameters and tumor vascularization.^5–9^ Micro-computed tomography (µCT) has high resolution and can be combined with capillary filling to investigate the microvasculature.^9^,^10^ Intravital laser scanning microscopy and multiphoton microscopy also offer high spatial resolution (∼200 nm) but are limited by a penetration depth of ∼500 µm and small field of view.^10^

Recently, selective plane illumination microscopy (“ultramicroscopy”) in conjunction with tissue clearing has gained significant interest. Several techniques have been developed that use fluids and colloids with the refractive index of proteins to render tissue fully transparent.^11–15^ Tissue clearing of entire organs or even organisms has recently been demonstrated and is becoming an essential tool in the life sciences and especially neuroscience community for circuit reconstruction and the study of 3D cellular distributions.^16^,^17^ Light sheet microscopy speeds up the acquisition of large datasets compared to confocal techniques and resulting datasets can encompass several gigabytes that can be recorded in a reasonable time. We have previously described an approach for assessing glioma microvessels in a mouse glioma model^18^,^19^ and have shown that this approach is also feasible in human glioma specimen.^20^ These approaches were, however, limited to quantifications of small tissue blocks and did not encompass the entire dataset due to lack of segmentation and post-processing tools.

In the present work, we present a numerical pipeline developed for the automated processing and analysis of large bio-imaging datasets with a software implementation usable with conventional hardware. We developed a Matlab program for the quantification of vascular networks in arbitrary 3D imaging volumes upon segmentation with specially trained classifiers in ilastik^21^,^22^ and automated macro-implementations for Fiji^23^ for pre-processing steps. The presented imaging pipeline enables large-scale, in-depth studies of vascular architecture in entire organs without the need for histological sectioning or advanced computer hardware.

## Methods

### Animal models

To assess the vessel architecture in a tumor context we injected 7.5 × 10^4^ U87-MG cells in nine weeks old, male NOD Scid Gamma (NSG; Jackson Laboratories, Bar Harbor, USA; *n* = 6 mice). Cells were injected into the right basal ganglia as described previously.^24^ Mice were sacrificed for imaging 21 days post tumor cell implantation. We compared intratumoral vessel morphology with nontumor bearing healthy control mice (nine weeks old, male NSG; *n* = 3 mice with six analyzed brain hemispheres).

For intravital dye labeling of the vasculature, mice were anaesthetized with ketamine 10% (90 µg/g bodyweight) and xylazinhydrochloride 2% (7.5 µg/g bodyweight) and injected intravenously with 100 µL of Texas red lycopersicon esculentum (Tomato) lectin (12 mg/kg, Vector laboratories TL-11,761 mg/mL). After 5 min of circulation, mice were transcardially perfused in deep anesthesia using 20 mL PBS followed by 20 mL 4% PFA. Embryos and whole brains were harvested and fixed overnight in 4% PFA, followed by PBS.

To study vessel morphology in development, we used three months old, female, pregnant C57BL6/6N mice (in house breeding at DKFZ) and extracted the embryos at E13.5 after injection with lectin of the mother animal in deep ketamine and xylazine hydrochloride anesthesia (*n* = 4 embryos) using the protocol described above. All experiments were approved by the regional animal welfare authority (Regierungspräsidium Karlsruhe, animal protocols: G127/16; DKFZ383) and were in accordance with the Federation for Laboratory Animal Science Associations (FELASA, category B) and Society for Laboratory Animal Science (GV-Solas, standard guidelines) and with the Guide for the Care and Use of Laboratory Animals published by the U.S. National Institutes of Health. Reporting complies with the ARRIVE guidelines (Animal Research: Reporting in Vivo Experiments).

### Clearing and imaging

Embryos and brains were cleared using the FluoClearBABB protocol over several days.^25^ In brief, samples were dehydrated using an ascending buthanol series from 30 to 100% (pH adjusted) for 24 h each. Embryos were transferred into BABB (pH-adjusted) and incubated for 48 h. After setting the refractive index (RI) of tissue to the RI of the clearing solution, samples became transparent. For imaging, a selective plane illumination microscope was used (Ultramicroscope II, LaVision Biotec, Bielefeld, Germany). Overview images of embryos were performed using 1x magnification (3.25 µm in-plane resolution). For magnifications of the vessel architecture of the embryo brain we used up to 4× magnification (800 nm in-plane resolution). For adult brains, a magnification of 1× was used (in-plane resolution of 3.25 µm) in order to acquire the entire brain, avoiding the necessity of stitching. For all experiments, the step size between image acquisitions in the transverse plane was 5 µm.

### Image analysis

Masks were manually drawn over the raw image data to delineate different regions to be analyzed individually and saved in equal-sized TIFF-stacks. The imaged vasculature was segmented from the raw images using the interactive learning and segmentation toolkit “ilastik”.^22^ Random forest classifiers in ilastik were trained simultaneously on all datasets from each cohort using a standard desktop PC. The trained classifiers were used on the datasets in an automated fashion using batch-processing in ilastik to export the binary vessel segmentations. The resulting vessel structures were smoothed with a 3D-Gaussian filter with isotropic σ = 1 (voxel units) using the 3D-smoothing plugin in the ImageJ distribution Fiji 2.0.0-rc-43/1.51r.^26^ Subsequently, the smoothed data were binarized with threshold at half of the voxel value range. A custom-written Matlab script was used to fill hollow vessels and holes in the binary vessel representations (Matlab version R2016b, Mathworks, Natick, MA, USA). Vessel centerlines were extracted with a custom-written macro using the skeletonization plugin in ImageJ.^27^ Branching points, vessel endpoints, and intermediate vessel skeleton voxels were automatically identified using the AnalyzeSkeleton plugin in ImageJ.^4^

### Automated quantification

Custom codes were written in Matlab to automatically quantify vascular parameters contained within an imaged volume of arbitrary shape, size, and resolution. To accelerate the quantification of large datasets and enable incremental analyses of large datasets, the developed program can dissect a given volume into cuboids of chosen dimensions. This allows for asymptotic studies of arbitrarily large acquisition volumes. Using different masks, independent regions of a segmented dataset can be analyzed individually.

Each subvolume from the 3D tiling box layout of chosen dimensions, imposed on the masked image data, is quantified with basic geometric measures in physical units. This includes the fraction of blood vessel volume in tissue, *fVV*, the microvascular density *MVD* (branch segments per mm^3^ tissue volume), and the vessel surface and length densities, *ρ_A_* (mm^2^ lumen area per mm^3^ tissue volume) and *ρ_L_* (mm vessel length per mm^3^ tissue volume), for a basic assessment of tissue perfusion density in each subvolume. The vessel segments between branching and/or endpoints are separately quantified as tubular objects; mean radius $\bar{r}$, segment length *l*, Euclidean vessel endpoint separation *d*, lumen surface area *A*, and tortuosity τ *= l/d*.^28^ Through an a priori labeling of each vessel branch in the original, undissected dataset, the vessel properties determined from different partitioning subvolumes are matched and combined to deliver estimates of the geometric properties of actual vessel branches without artificial divisions. The quantification algorithm is tailored to treat vascular networks with many small, interwoven structures sized close to the pixel resolution and has been validated with well-defined ground truth models for precision (see Supplemental methods and Supplemental Figure 1 for further details).

**Figure 1.**
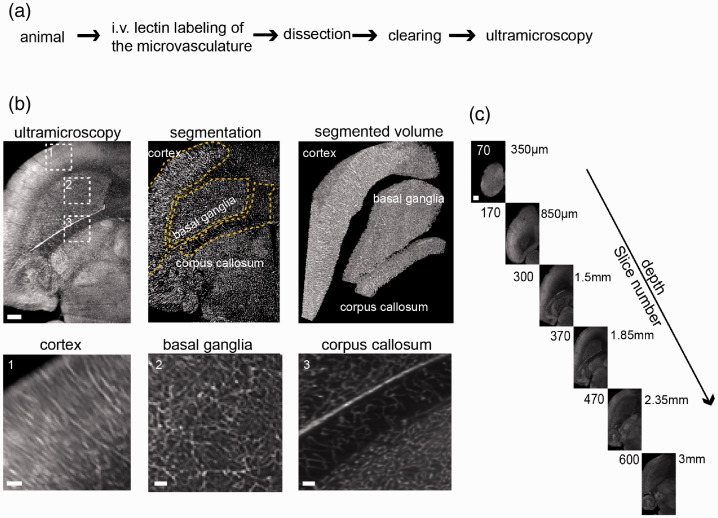
Ultramicroscopy of the microvasculature in healthy mice. (a) Experimental outline for tissue preparation and SPIM. Healthy, female black six wild-type mice were injected intravenously with fluorescent lectins to label the microvasculature before perfusion. (b) Ultramicroscopic image processing and three-dimensional view of the analyzed regions of interest in the healthy mouse brain, magnified images (dashed boxes) of cortex, basal ganglia, and corpus callosum. Yellow dashed lines indicate the segmented subregions. (c) Images of the acquired z-stack. Step size is 5 µm. The entire stacks consisted of ∼600–1000 single-plane images. Scale bar = 500 µm and 100 µm in magnified images in B.

Parameter distributions characterizing the geometric vessel properties of given datasets are saved for the vasculature in the individual subvolumes and automatically combined to deliver a global quantification of the tissue regions marked by the masks. The geometric characteristics of individual vessel segments are saved in lists with matched entries of $\bar{r}$, *l*, *A*, and τ. These lists can be processed further for statistical analyses. The processing order of the partitions from the 3D grid can be chosen sequentially or randomly and already analyzed subvolumes can be merged for combined statistics while the basic analysis of individual subvolumes is still running in parallel. This offers the possibility to analyze parameter distributions statistically before an image was entirely processed. This can be useful with large datasets and limited computational power. Analyses can be restarted and will continue in case of a system failure or limited computational time slots, making our pipeline useful for processing large datasets. The developed numerical pipeline including validation scripts and a user documentation are available from the authors upon reasonable request.

### Statistical analysis

Separate Matlab codes were developed to process the extracted results, conduct statistical testing and provide data visualizations. Vessel property distributions and parameters extracted from the sampled subvolumes were compared between different tissue groups. Statistical hypothesis testing between groups was performed with the nonparametric Kruskal–Wallis test in Matlab, using mean parameter values from each specimen. We used nonparametric tests because sample size of *n* = 3 to 6 animals was too small to test for normal distribution. Histograms and 2D-distribution representations can automatically be created with optional discrimination of vessel segments in certain radius-, length-, or tortuosity-ranges. Data are presented as mean ± standard deviation (S.D.) and *p* < 0.05 was considered significant (*p < 0.05; **p < 0.01; ***p < 0.001).

## Results

For dissecting the microvascular anatomy in its entirety, we employed an intravital dye labeling approach using intravenously injected fluorescent lectins.^29^ Lectins were injected, animals sacrificed and cleared using the FluoClearBABB protocol (Figure 1(a)). After clearing, imaging was performed by selective plane illumination microscopy. Labeled vessels were segmented semi-automatically in the entire dataset based on edge and texture features and fluorescence intensity. We analyzed various vessel parameters including vessel density, segment length, radius, tortuosity, surface area, and ratio of vessel volume within the tissue. The analysis generated data points for 5 × 10^4^ to 3 × 10^6^ vessel segments per sample. To demonstrate the utility of the approach, we probed microvascular features in development, physiological, and pathological conditions.

### Uncovering divergent microvascular architectures in different brain regions

**Figure 2.**
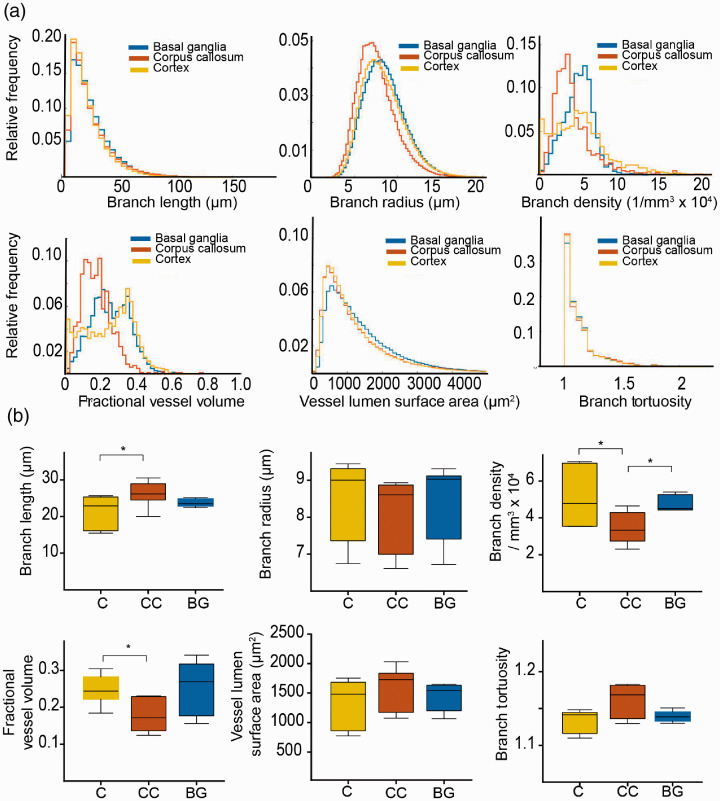
Microvascular parameters in the healthy mouse brain. (a) Histograms show quantification results from ultramicroscopic images and demonstrate significant differences between the indicated vessel parameters in different regions of the healthy brain. (b) Whisker plots demonstrate vessel parameters of different regions in the healthy brain (median with 50% quantile and extremal values (whiskers) from *n* = 3 mice for six analyzed brain hemispheres. * =*p*<0.05. C: cortex; Cc: corpus callosum; BG: basal ganglia.

Healthy mice were injected with lectin-FITC, sacrificed and cleared. After clearing of the whole brain, both hemispheres were recorded by ultramicroscopy (*n* = 6 hemispheres from *n* = 3 mice). In order to investigate microvascular parameters in different regions of the healthy brain, we extracted vascular parameters in the cortex (grey matter), corpus callosum (white matter), and basal ganglia using masks and counter masks (Figure 1(b) and (c), Supplemental movie 1, 2). Each brain region contained distinct vascular properties: The cortex yielded highest variance in microvascular density (*MVD)* (Figure 2(a)). Overall, the mean *MVD* in the corpus callosum was markedly lower (3.5 ± 0.9 × 10^4^ branch vessels/mm^3^) compared to the cortex (5.1 ± 1.6 × 10^4^ mm^−3^, *p* < 0.05) and basal ganglia (4.8 ± 0.5 × 10^4^ mm^−3^, *p* < 0.05, Figure 2(a) and (b)). Also, the corpus callosum showed lower mean partial vessel volumes *fVV* (corpus callosum; 0.18 ± 0.05 vs. cortex; 0.25 ± 0.04, *p* = 0.01) and longer vessel segments (corpus callosum; 26.2 ± 3.5 µm vs. cortex; 21.43 ± 4.5 µm, *p* = 0.02). Vessel segments in the corpus callosum also showed a trend towards higher tortuosity compared to the other regions (corpus callosum; 1.16 ± 0.03 vs. cortex; 1.13 ± 0.02 and basal ganglia; 1.14 ± 0.01, *p* > 0.05). Similarly, while the mean vessel surface density *ρ_A_* did not differ significantly (corpus callosum; 36 ± 23 mm^−1^ vs. basal ganglia; 41 ± 10 mm^−1^ and cortex; 38 ± 11 mm^−1^, *p* > 0.05), the vessel length density *ρ_L_* underlined the reduced vascular proliferation in the corpus callosum (corpus callosum; 534 ± 95 mm^−2^ vs. basal ganglia; 793 ± 75 mm^−2^, *p* = 0.004 and cortex; 737 ± 96 mm^−2^, *p* = 0.01). Mean vessel radius and surface area did not differ significantly between the examined brain regions (*p* > 0.05, Figure 2(a) and (b)).

### Quantitative assessment of tumor angiogenesis

**Figure 3.**
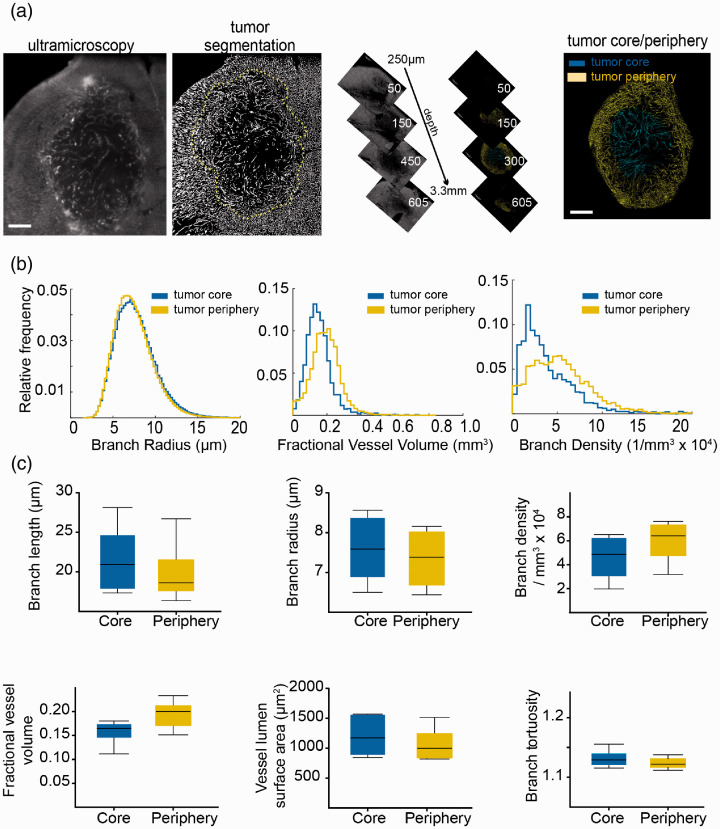
Microvasculature remodeling in the U87 glioma model. (a) Ultramicroscopic image processing and three-dimensional view of region of interest in mouse U87 glioma tumors. Yellow dashed line indicates the segmented tumor area. (b) Histogram representation of quantified vessel parameters showing tumor core and tumor periphery. (c) Whisker plots are shown for the obtained parameters of U87 mouse tumors (*n* = 6 mice). Scale bar = 500 µm.

To investigate microvascular dynamics in a pathological paradigm, we employed the U87-MG glioma model (*n* = 6 mice). After lectin labeling of the microvasculature and clearing, tumors were divided into two separate tumor compartments: the tumor periphery, representing the infiltrative zone (outer 50% of the tumor, as measured from the centroid of the mask in the radial direction) and the tumor core (inner 50%). We hypothesized that the tumor core might show major microvascular differences compared to the tumor periphery (Figure 3(a), Supplemental movie 3). However, contrary to our hypothesis, most vessel parameters did not differ significantly between the tumor core and tumor periphery (Figure 3(b) and (c)), except the fractional vessel volume *fVV* (tumor core; 0.16 ± 0.03 vs. tumor periphery; 0.20 ± 0.03, *p* = 0.05). The mean vessel length density *ρ_L_* (tumor core; 511 ± 143 mm^−2^ vs. periphery; 633 ± 164 mm^−2^, *p* = 0.2) and surface density *ρ_A_* (tumor core; 30 ± 15 mm^−1^ vs. periphery; 34 ± 17 mm^−1^, *p* > 0.05) did also not differ significantly.

### Probing the microvasculature in development

**Figure 4.**
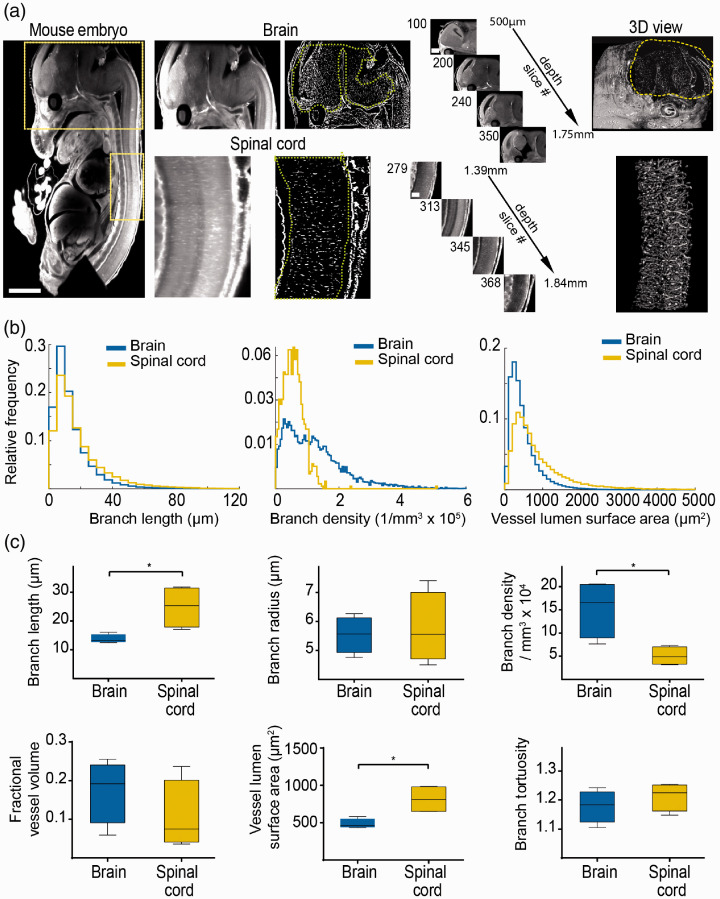
Assessing microvascular parameters in development. (a) Ultramicroscopic image processing and three-dimensional view of the brain and spinal cord in the mouse embryo (e13.5). Yellow boxes indicate magnified regions (brain and spinal cord). (b) Histogram representations of the quantified ultramicroscopic images demonstrate significant differences in vessel parameters between brain and spinal cord. (c) Whisker plots show parameter statistics for the spinal cord and brain microvasculature (*n* = 4 embryos). Scale bar is 500 µm in embryo images, 250 µm in magnified brain images, and 100 µm in magnified spinal cord images. * = *p*<0.05.

To further extend our approach and show the applicability to developmental studies, we assessed the microvasculature in embryonic mice (e13.5, *n* = 4 embryos). Intravenous lectin injection of the mother animal resulted in excellent labeling of the entire embryonic microvasculature, including microvessels in the nervous system, parenchymal organs (e.g. heart, liver, kidney), and cardiovascular system with high signal-to-noise ratio (Figure 4(a), Supplemental movie 4, 5). We focused our analysis on the central nervous system and compared microvascular properties of the brain and spinal cord **(**Figure 4(a) to (c)). Analysis of vessel parameters demonstrated that the spinal cord exhibits significantly longer vessel segments with less branches compared to the developing brain (mean vessel segment length, spinal cord; 24.6 ± 7.8 µm vs. embryonic brain; 13.8 ± 1.6 µm, *p* < 0.05, Figure 4(c)). This resulted in a larger vessel surface area in the spinal cord compared to the brain (spinal cord: 775.6 ± 150.6 µm^2^ vs. embryonic brain; 483.6 ± 66.5 µm^2^, *p* = 0.02). Furthermore, the mean vessel density was significantly lower in the embryonic spinal cord compared to the embryonic brain (spinal cord; 5.4 ± 2.3 × 10^4^ vs. brain; 15.3 ± 6.3 × 10^4^, *p* = 0.02). While the vessel length density *ρ_L_* exhibited a wide distribution (spinal cord; 313 ± 246 mm^−2^ vs. brain; 475 ± 306 mm^−2^, *p* > 0.05), the mean vessel surface density *ρ_A_* showed significant differences (spinal cord; 28 ± 6 mm^−1^ vs. brain; 47 ± 8 mm^−1^, *p* = 0.02). The mean vessel radius, tortuosity, and fractional vessel volume showed no significant differences between the embryonic brain and spinal cord (Figure 4(c)).

## Discussion

Vascular patterning is crucial for understanding embryonic development, growth, and pathology. Cancer is one of the most notable examples, in which vessel parameters influence the prognosis and treatment outcome.^30^ Neo-angiogenesis has been attributed as a main denominator for tumor progression and metastasis. This has led to an increased interest in employing inhibitors of angiogenesis to curb cancer and antiangiogenic agents have become standard of care for, e.g. ovarian cancer or renal cell carcinoma.^31^ In addition, there is also ample evidence to suggest a crucial role of vasculature parameters for the success of chemotherapeutic and immunotherapeutic treatment regimens.^32^ Paradoxically, in other tumor contexts, antiangiogenic treatments have been linked to earlier progression, increased invasion, and metastasis formation.^33^ Impaired microvascular function may lead to tumor hypoxia, a crucial factor associated with the development of resistant cell populations.^34^ Therefore, further investigations into mechanisms of angiogenesis and possible druggable targets in preclinical models are warranted.^35^

Ultramicroscopy and tissue clearing using light sheet microscopy have gained a lot of traction in recent years, especially in the field of neuroscience.^17^ However, despite the unprecedented 3D resolution, the utility of the technology has been mainly limited to qualitative studies.^36^,^37^ The quantitative analysis of ultramicroscopy datasets was significantly hindered by the lack of analyses protocols and tools to segment all objects of interest in the complex and large 3D datasets that can easily encompass several gigabytes from a single acquisition. In our study, the analysis of each sample generated, on average, 5 × 10^4^ (in embryonic spinal cord) to 3 × 10^6^ single data points (in healthy brain samples). The technique employed by us can analyze vessel radii as small as 3 µm, limited by the image resolution, and robustly generates the main morphological microvascular parameters without the need for high-performance computational hardware or expensive proprietary licenses for image processing software (with the exception MATLAB).

To demonstrate the utility of our platform, we analyzed whole embryos as well as healthy and tumor containing brain samples. The technique is also amenable to other organs with good labeling efficacy (Supplemental Figure 2). After clearing, microscopic image acquisition of a tissue sample took ∼4 h. We employed ilastik^22^ to segment the entire vasculature contained in the image stacks, which took 30–90 min for classifier training and ∼4–6 h for automated pixel classification and TIFF-export of an image stack of 1 GB in an 8-bit format (1766 × 1284 × 446 (∼10^9^) pixels). The segmented “angiome” was quantified using custom-written MATLAB code designed to analyze and calculate the length of vessels between branches, radius, tortuosity, surface area, and various vessel density parameters. The automated quantification of a 1 GB dataset containing 200,766 vessel branches took ∼1 h with a standard, quad-core computer and 16 GB of random access memory (RAM). The entire processing pipeline can be executed on a standard personal computer, with the feasible image size being limited only by the RAM addressable by ilastik and MATLAB.

Our developed processing algorithm enables incremental analyses of large datasets, processing 3D data in a partitioned manner and saving partial results. If the MATLAB quantification is interrupted, the code can be restarted with identical settings to continue where it left off, sparing already quantified subvolumes. This makes “asymptotic analyses” possible. If appropriate for a given investigation, long analyses can be terminated once the parameter distributions reach an asymptotic state, where further processing of more randomly sampled subvolumes is not expected to change relative parameter distributions. Such methods may become relevant for future investigations such as, e.g. large-tissue µCT or high-resolution, large field-of-view imaging modalities.

Our analysis of different areas of the healthy brain demonstrated a significant difference in several microvascular parameters, such as vessel density, tortuosity and partial vessel volume between cortex, basal ganglia, and corpus callosum. Our analysis demonstrated that white matter regions like the corpus callosum have longer vessel segments with less branches and lower vessel density, consistent with lower energy demands of long-range axonal projections compared to grey matter regions of the cortex and basal ganglia.^38^ This finding was mirrored in the embryonic spinal cord when compared to the embryonic brain where branch length and branch density in the spinal cord was also lower compared to the brain.

The analysis of U87 tumors was performed on the tumor core versus tumor periphery. In contrast to our hypothesis that the infiltrative tumor border might have increased vascular densities and more aberrant vessel morphologies compared to the tumor core, there were only minor differences between the two tumor regions, except for the mean fractional vessel volume, which was higher in the tumor periphery. The absence of significant differences between the other vessel parameters might be explained by the lack of necrosis and rather uniform vascular patterning in U87 tumors at the stage of analysis (day 21 after tumor cell implantation). It is well conceivable that pathological angiogenesis kicks in only at later tumor stages (e.g. day 35 after tumor cell implantation) or is more prominent in other, more invasive brain tumor models.^39^

We further demonstrate the value of our approach for developmental studies in mice embryos. We found significant differences between microvasculature patterns of different organs in the embryo. Our analysis of the microvasculature of brain and spinal cord revealed differences in vessel branch densities consistent with the idea that the higher neuronal density in the brain requires higher vascular supply. The spinal cord consisted of longer vessel segments with fewer branches compared to the brain, presumably because of the long fiber tracts and lower neuronal densities in the spinal cord compared to the brain.

The quantitative analysis presented here constitutes a proof-of-principle study to showcase the developed pipeline. The data processing and analysis can be easily adapted to different experimental setups. Limitations of our study include the necessity to obtain high-quality optical recordings with high signal-to-noise ratio in order to achieve robust segmentations. We found that the trainable toolkit “ilastik” provided the best segmentation of our data, offering flexibility and customization to suit different image types, and did not demand excessive processing power, but the segmentation step can be substituted with a method of choice. Our technique includes semi-automatic segmentation, data post-processing, and comprehensive quantitative analyses. By using a partitioning approach, the analysis of an arbitrarily formed and sized 3D image segmentation can be conducted on standard desktop and laptop computers without the need for advanced computing resources, such as computing clusters or graphics cards.

While there are different image post processing tools available, such as Imaris or Amira to visualize, analyze, and quantify microscopy data, our processing pipeline was optimized to quantify complex vascular structures with microvessels sizes at the scale of the image resolution. With a vessel radius estimation tailored to the microvascular scale and connectivity, as well as the entirely automated quantification of an extensive set of basic geometric parameters, our pipeline is able to efficiently analyze vascular datasets. While Imaris and Amira offer powerful capabilities for data visualization, animation, and interactive exploration, their generality and ability to deal with large input images is a disadvantage in comparison to our pipeline. Such powerful image processing tools allow manual data exploration but could in our hands not characterize thousands to millions of vessels in a given dataset.

The vessel radii found in this study and consequently also fractional vessel volumes are most likely overestimated by the imaging procedure and limited resolution inherent to our experimental setup. Since most capillaries are on the order of one to two pixels, the overlaying point spread function leads to an “over-estimation” of the segmentation, manual, and automated alike. At an axial resolution of 3–5 µm, this can amplify capillary radii and vessel volumes, leading to artificially large values. This constitutes a systematic error throughout the experiment. Additional post-processing steps, e.g. morphological thinning of the segmented images can be applied for correction.^18^ Statistical analysis of the presented data is difficult to perform due to the wealth of data: we compared the mean of each parameter per mouse, thus averaging thousands of data points per animal in order to not overestimate effect sizes in our statistical analysis. Our approach could further be extended to radiomics and atlas-based registration as previously highlighted^40^ and different other quantitative measures can be implemented in the piecewise analysis, such as local connectivity and regularity parametrizations.^18^

In summary, ultramicroscopy is a fast and straightforward technique that generates large datasets of entire organs or organisms. Our described “toolbox” can be used to investigate physiological and pathological states as well as treatment regimens in disease models. Imaging of the microvasculature using SPIM served as a proof of principle; our processing pipeline can be applied to 3D data from any imaging modality, e.g. µCT, magnetic resonance angiography, laser-scanning, or electron microscopy. Our approach can obtain robust and quantitative microvascular parameters, which could be easily expanded to other fluorescent objects of interest that can be quantified in a partitioned manner, e.g. immune cell distributions, neuronal projections, or migratory patterns of fluorescently labeled stem or tumor cells to quantitatively map cellular 3D distributions in entire organs and intact organisms.

## Supplemental Material

sj-pdf-1-jcb-10.1177_0271678X20961854 - Supplemental material for Large-scale characterization of the microvascular geometry in development and disease by tissue clearing and quantitative ultramicroscopy Click here for additional data file. Supplemental material, sj-pdf-1-jcb-10.1177_0271678X20961854 for Large-scale characterization of the microvascular geometry in development and disease by tissue clearing and quantitative ultramicroscopy by Artur Hahn, Julia Bode, Allen Alexander, Kianush Karimian-Jazi, Katharina Schregel, Daniel Schwarz, Alexander C Sommerkamp, Thomas Krüwel, Amir Abdollahi, Wolfgang Wick, Michael Platten, Martin Bendszus, Björn Tews, Felix T Kurz and Michael O Breckwoldt in Journal of Cerebral Blood Flow & Metabolism

sj-pdf-2-jcb-10.1177_0271678X20961854 - Supplemental material for Large-scale characterization of the microvascular geometry in development and disease by tissue clearing and quantitative ultramicroscopy Click here for additional data file. Supplemental material, sj-pdf-2-jcb-10.1177_0271678X20961854 for Large-scale characterization of the microvascular geometry in development and disease by tissue clearing and quantitative ultramicroscopy by Artur Hahn, Julia Bode, Allen Alexander, Kianush Karimian-Jazi, Katharina Schregel, Daniel Schwarz, Alexander C Sommerkamp, Thomas Krüwel, Amir Abdollahi, Wolfgang Wick, Michael Platten, Martin Bendszus, Björn Tews, Felix T Kurz and Michael O Breckwoldt in Journal of Cerebral Blood Flow & Metabolism

sj-pdf-8-jcb-10.1177_0271678X20961854 - Supplemental material for Large-scale characterization of the microvascular geometry in development and disease by tissue clearing and quantitative ultramicroscopy Click here for additional data file. Supplemental material, sj-pdf-8-jcb-10.1177_0271678X20961854 for Large-scale characterization of the microvascular geometry in development and disease by tissue clearing and quantitative ultramicroscopy by Artur Hahn, Julia Bode, Allen Alexander, Kianush Karimian-Jazi, Katharina Schregel, Daniel Schwarz, Alexander C Sommerkamp, Thomas Krüwel, Amir Abdollahi, Wolfgang Wick, Michael Platten, Martin Bendszus, Björn Tews, Felix T Kurz and Michael O Breckwoldt in Journal of Cerebral Blood Flow & Metabolism
