# Supplementary figures and images for: Large-scale characterization of the microvascular geometry in development and disease by tissue clearing and quantitative ultramicroscopy
Source: J Cereb Blood Flow Metab. 2020 Oct 12;41(7):1536–46. doi: 10.1177/0271678X20961854 (PMC8217891; doi:10.1177/0271678X20961854)

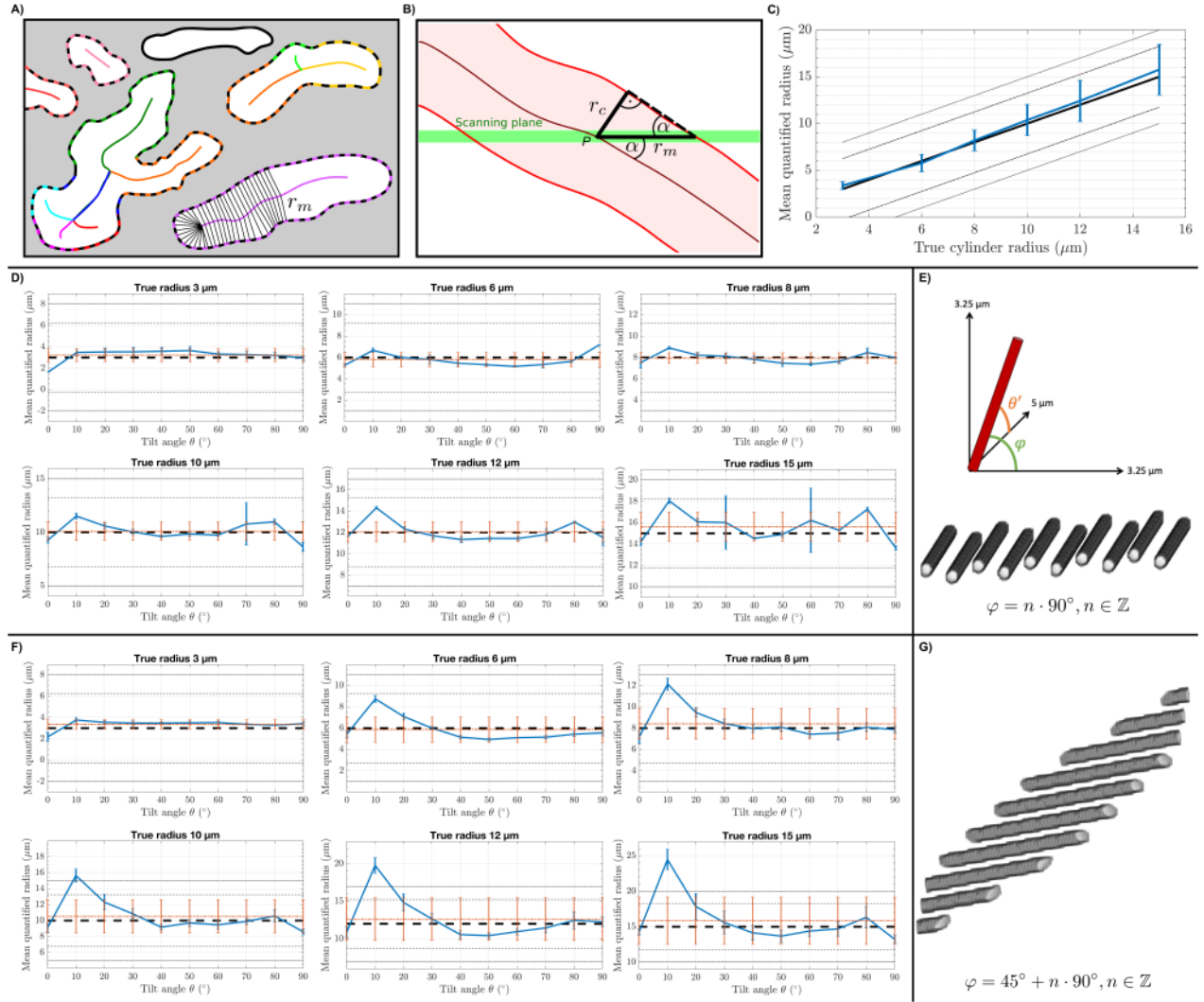

Suppl. Fig. 1, Quantitative ultramicroscopy, Hahn et al.,

Supplement: sj-pdf-1-jcb-10.1177_0271678X20961854 - Supplemental material for Large-scale characterization of the microvascular geometry in development and disease by tissue clearing and quantitative ultramicroscopy [file sj-pdf-1-jcb-10.1177_0271678X20961854.pdf]

A

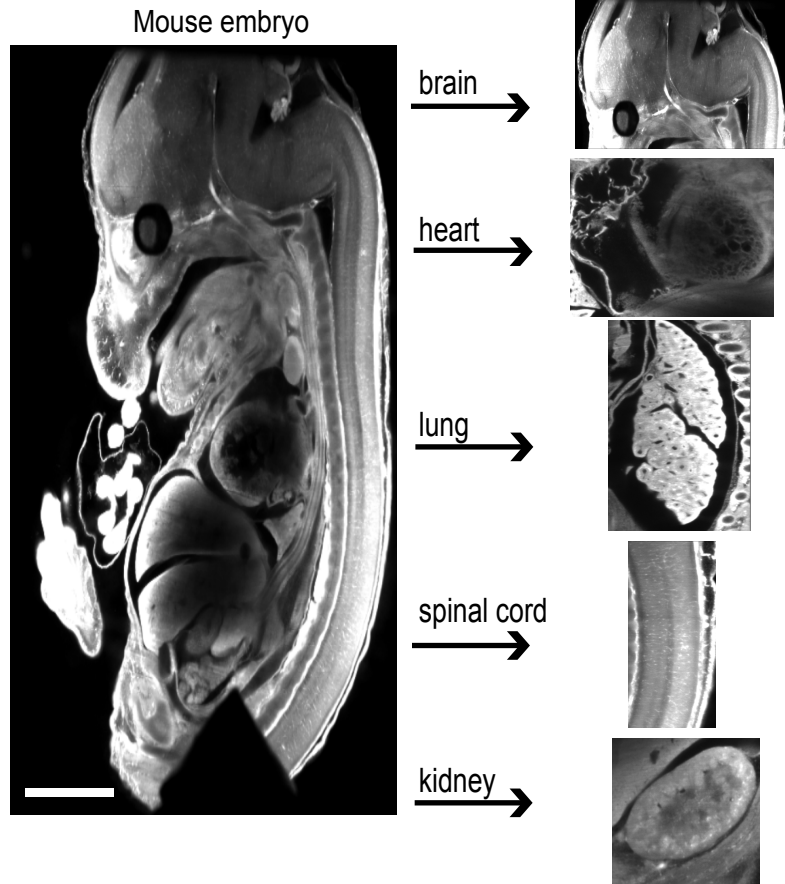

B

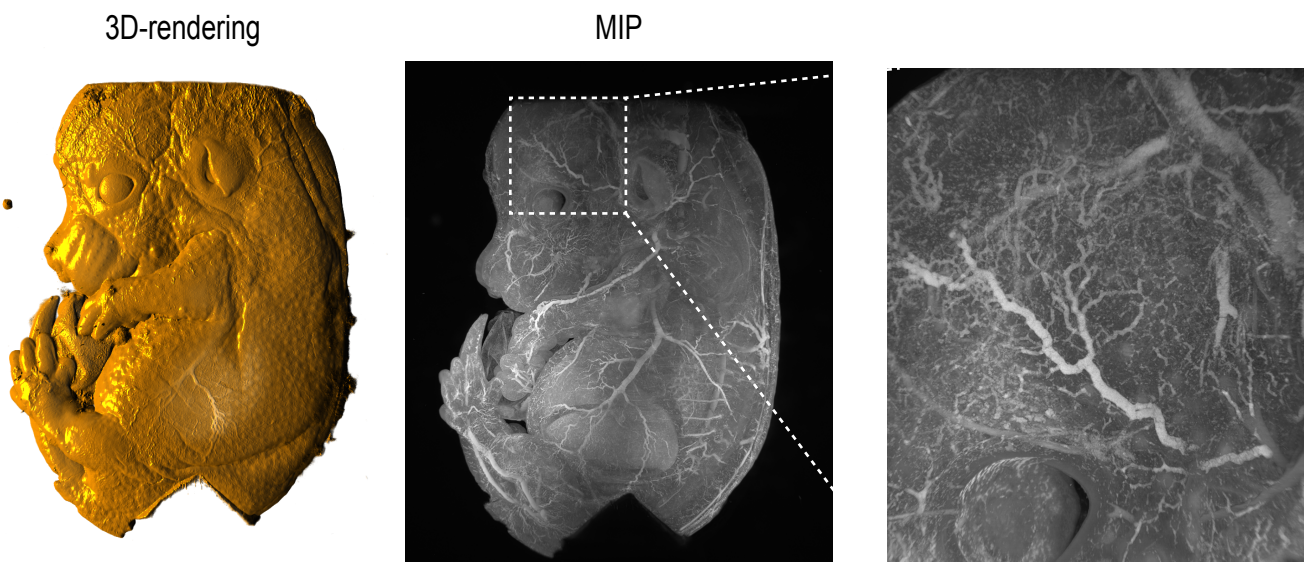

Suppl. Fig. 2, Quantitative ultramicroscopy, Hahn et al.,

Supplement: sj-pdf-2-jcb-10.1177_0271678X20961854 - Supplemental material for Large-scale characterization of the microvascular geometry in development and disease by tissue clearing and quantitative ultramicroscopy [file sj-pdf-2-jcb-10.1177_0271678X20961854.pdf]
