# Supplementary material for: Large-scale characterization of the microvascular geometry in development and disease by tissue clearing and quantitative ultramicroscopy
Source: J Cereb Blood Flow Metab. 2020 Oct 12;41(7):1536–46. doi: 10.1177/0271678X20961854 (PMC8217891; doi:10.1177/0271678X20961854)
Supplement: sj-pdf-8-jcb-10.1177_0271678X20961854 - Supplemental material for Large-scale characterization of the microvascular geometry in development and disease by tissue clearing and quantitative ultramicroscopy [file sj-pdf-8-jcb-10.1177_0271678X20961854.pdf]

Supplemental information: Hahn et al.,

Supplemental figure captions:

Supplemental Fig. 1: Quantification details and validation of determined vessel radii. **A)** Sketch of a schematic 2D scanning plane with several vessels cutting the plane (the tissue background is colored in gray). The central top vessel area (with black outline) does not contain a skeleton segment in the depicted slice and is therefore ignored in this slice. The vessel cuts containing labeled skeleton pixels are processed by iterating over the perimeters of each area, marked by dashed black-colored lines, matching the label color of the nearest contained skeleton branch. As demonstrated in half of the bottom right vessels (purple), for each pixel on the perimeter of the cross section, the distance to the nearest skeleton pixel is determined (considering the real physical pixel resolution, making this approach stable even for very anisotropic pixel dimensions). In cases where differently labeled skeleton branches lie within one vessel cross section, the perimeter is processed piecewise with pixel distances assigned to their closest branch (see top right and bottom left cross sections). Each radius value determined within a scanning plane (x-y, x-z, and y-z plane), called r_m, is projected onto the plane orthogonal to the local 3D skeleton orientation. **B)** Sketch of a scanning plane cutting a vessel as seen from above, with angle α between the skeleton and the scanning plane at skeleton pixel *P*. If a segment is not parallel to the imaging plane, the measured radius r_m is multiplied with $\sin(\alpha)$ for a corrected radius r_c, approximating the true radius orthogonal to the vessel orientation. The r_c values attained from all perimeter pixels along each scanning direction are averaged for each branch label, which yields mean segment radii based on the lumen shape. **C)** Accuracy validation summary of the implemented radius quantification from analyzing model volumes with differently oriented cylinders with pre-defined radius. The true cylinder radius is on the x-axis and the radii quantified by the algorithm are shown as mean and standard deviation over all tested vessel orientations (see panels **D** and **F** for details). The true radius is plotted in black beneath the blue error bar lines and a deviation of the in-plane resolution (3.25 µm) is marked by thin dashed lines, with the z-resolution range (5 µm) as thin dotted lines. Panels **D** and **F** present the mean quantified radii of cylinders with different orientations between the x and y axis (defined by the angle φ) and the z-axis (parametrized by θ, see panel **E**) and predefined radii of $r = \{3,6,8,10,12,15\}$ µm. The true radius is plotted as a bold dashed, horizontal line, while the quantified radii of ten identically oriented but differently positioned cylinders are presented for each angle θ between 0° and 90° through the mean and standard deviation among those ten cylinders with blue connected error bars. For each model radius, the mean determined radius and its standard deviation over the θ orientations is shown as orange error bars across the entire range. The pixel resolutions are shown, added to and subtracted from the true cylinder radius, by thin, dashed and dotted lines. **D)** The results for different θ orientations with $\varphi = 0°$, i.e. the cylindrical axis running parallel to the x-direction with 3.25 µm pixel size. **F)** The same analysis with cylinders oriented diagonally in the x-y-plane with $\varphi = 45°$. **E)** Visualization of the angles θ and φ with a 3D rendering of synthesized cylinders (generated by the Volume Viewer plugin in Fiji) with a radius of

8 µm at a resolution of 3.25 x 3.25 x 5 µm and $\theta = 10°$. **G)** Volume rendering of 8 µm validation cylinders with $\theta = 10°$ and $\varphi = 45°$ at our imaging resolution.

Supplemental Fig. 2: Different organs of the mouse embryo in ultramicroscopy that can be used for analysis and quantification (A). 3D rendering and maximum intensity projection of a mouse embryo after lectin FITC labeling of the mother animal (**B**).

Supplemental movies captions:

Suppl. movie 1: Ultramicroscopy of the healthy mouse brain. Raw images (left column) and segmented images (right column) are shown.

Suppl. movie 2: Healthy mouse brain regions (basal ganglia, corpus callosum, and cortex) that were used for quantification.

Suppl. movie 3: Ultramicroscopy of U87 tumor bearing mice with segmented SPIM images after clearing (left column) and segmented images after application of the mask (right column). Tumor periphery (pseudocoloured in yellow) and tumor core (pseudocoloured in blue) are shown.

Suppl. movie 4: Ultramicroscopy of the mouse embryo. Raw SPIM images after clearing (left column) and segmented images before (middle column) and after application of the mask (right column) are shown.

Suppl. movie 5: Ultramicroscopy of the mouse embryo spinal cord. Raw SPIM images after clearing (left column) and segmented images before (middle column) and after application of the mask (right column) are shown.

Supplemental methods:

Automated partitioning analysis

After the automated dissection of a segmented and post-processed input dataset, each subvolume of desired dimensions (in 3D) is analyzed individually. Different regions of interest (ROIs), marked by manually drawn masks on the original image data beforehand, are considered exclusively within the cuboid subvolumes, also with partial volume coverage. The subvolumes are analyzed consecutively, either sequentially following the linear index of the partitions, or in random order to allow for asymptotic analyses of very large datasets. The partitioned analysis can be terminated and picked up again where left off, by restarting the program with identical settings. Quantifications are conducted using the post-processed image stacks, containing the binary vessel architecture, as well as the tagged skeleton data with differentiated centerline voxels. The voxel size (resolution) along each imaging dimension needs to be specified and is used to provide results with physical units (in our study, on the scale of micrometers).

Before partitioning the masked image volume for quantitative processing, the original vessel segments in the undissected image are labeled. When the analysis of the

individual partitions is finished (or while it is still running, with some partitions finished), a separate script can be executed in a second Matlab instance to merge the results present thus far. The vessel properties determined in the individual partitions are combined to deliver estimates of true vessel segment properties without artificial cuts through processing. This is done by comparing the branch labels within each partition with the original, globally labeled data. Vessel lengths and surfaces are added, while tortuosity and mean segment radii are calculated by a weighted average using the partitioned segment lengths as weights. The finally determined vessel properties are written to lists in text files in matching order, with an additional text file showing the labels of the corresponding branches in the original dataset. Thus, the vessels corresponding to different labels can be located in the original dataset for visual analysis.

Perfusion density quantifications

Vascular density measures are determined for each subvolume of predefined dimensions. The fractional vessel volume *fVV* is given by the ratio of voxels identified as blood vessels to the total number of voxels within a region of interest in the cuboid, marked by the respective mask. The *MVD* is calculated as the number of individual vessel segments per mm^3 tissue volume within a masked region. Vessel segments are defined by the skeleton's branch voxels between two branching/end points.

The microvascular surface density ρ_A is quantified for each subvolume to give a measure of lumen surface area with respect to tissue volume under supply. The vessel surface area is determined in physical units of mm^2 using the discrete voxel intensity gradients along each imaging dimension. The discrete gradient is calculated by subtracting intensities of voxels with an offset of one voxel length along each dimension. Taking the absolute value, the resulting image stacks show vessel edges along each dimension in binary form. The edge information along each dimension is combined and linearly interpolated between voxels to attain the vessel surface as a tessellation of voxel surface elements with known physical area (see next subsection for details). The sum of all surface elements in a volume is normalized by the physical tissue volume in mm^3 to provide the vessel surface density ρ_A in mm^{-1}. The total Euclidean skeleton length is normalized with the tissue volume to provide the averaged vessel length density ρ_L for the region of interest in mm/mm^3.

Individual vessel quantifications

Individual vessel segments between branching points are labeled and analyzed geometrically within each processing partition. The segment length *l* is obtained as the sum of Euclidean inter-voxel distances along the vessel centerline between branching points and/or end points, considering physical voxel dimensions. The segment tortuosity τ is simply characterized by the ratio $\tau = l/d$, with the geodesic endpoint separation [illegible] [1]. This dimensionless quantity is often called the distance metric and gives a simple quantification of the degree of curvature of a vessel segment [2].

The mean radius $\bar{r}$ of a vessel segment is determined as the average over all radius values [illegible] along its skeleton line. For each voxel on the centerline of a branch, the radius $\bar{r}$ at that point is estimated using two methods. The first method is a simple implementation using Euclidean distance maps, generated with the Matlab-function 'bwdist' [3]. For each skeleton point, the nearest background voxel in the binary vessel dataset is identified to determine the corresponding physical distance using the voxel resolution. This works best for isotropic resolutions, since the distance map does not consider physical pixel sizes. This method can be deactivated with a Boolean flag in the script if the image resolution is too anisotropic. In this case, only the following method is used to estimate the mean segment caliber.

A second radius value for each skeleton point is estimated using the lumen surface of the vessels. Along each dimension, 2D-slices of the imaged volume are consecutively scanned and vessel boundary voxels, surrounding each skeleton segment, are probed to average their physical distance from the centerline. The distances in the standard scanning slices (x-y-, x-z, and y-z plane) are projected onto the plane perpendicular to the 3D orientation of the skeleton segment at the respective point of intersection with the scanning plane using a trigonometric correction with $\sin(\alpha)$, where α is the angle between the local 3D vessel skeleton orientation and the scanning plane at the point of intersection *P* (see new **supplemental Fig. 1 A,B**). This correction provides an estimate of the radius values perpendicular to the vessel centerline. In each scanning slice, the cut vessels with at least one skeleton pixel inside are processed to attain a radius estimate from every pixel of the respective vessel cross section's perimeter (see **supplemental Fig. 1 A,B** for details). From all three scanning directions, the values accumulated for each vessel segment are averaged to attain an estimate of the segment's mean radius.

The mean radius values from the second method are averaged with the radii from the distance map method with equal weighting, unless deactivated due to strong resolution anisotropy. The mean segment radius $\bar{r}$ is quantified as the average of all values determined for the respective vessel segment. During the second radius estimation method, the area of each vessel surface element is added up to quantify the lumen surface area [illegible] in μm^2. Each geometric property is associated with the respective segment label in a subvolume under analysis and saved in lists.

Vessel branches in the original input data were labeled before dissection. The properties of vessel instances divided by artificial dissection are determined by combining the corresponding vessel piece characteristics. The mean branch radius $\bar{r}$ and tortuosity τ are averaged, using the respective segment lengths in different partitions as weights; the length [illegible] and surface area [illegible] are summed for each vessel labeled in the original, masked image volume.

Evaluation of quantification accuracy

Most quantitative measures determined by the custom algorithm in Matlab are well-defined and straight-forward to calculate numerically (see previous subsections), given a valid segmentation of the imaged vasculature. For realistic vessel networks with tortuous segments and complex connectivity, the mean radius is an ill-defined property, which is non-trivial to determine. Initially, we tried implementing a radius quantification using virtual planes, oriented perpendicular to the 3D skeleton at each pixel of the skeleton with different interpolation techniques and corrections to project and discern individual vessel parts in arbitrarily placed planes through the discrete image volume. This turned out to be problematic due to the interpolation of structures sized near the resolution limit, thus we restarted with an entirely new approach avoiding interpolation, specifically tailored to data with many relevant structures around the image resolution (see previous subsection for details).

The radius quantification we present in this work was validated using synthetic TIFF-stacks created with a custom Matlab script, holding “segmented” cylinders with well-defined radii at different 3D orientations and pixel dimensions of 3.25 x 3.25 x 5 µm. Each synthesized volume contained 10 straight cylinders with identical 3D orientation and different positions, but no intersections in order to avoid variable radii at crossings (see **supplemental Fig. 1, E,G** for examples and relevant orientation angles). The “polar” angle θ between the original imaging plane (3.25 µm resolution) and the orthogonal z-axis in stack direction (5 µm steps) was varied in steps of 10° between 0° and 90°, covering the non-periodic range. The in-plane angle φ between the x- and y-axes was set to 0° and 45° to cover the two in-plane orientations that yield the highest difference in effective pixel lengths of the in-plane axis perpendicular to the cylinder orientation (3.25 µm and $\sqrt{2 \cdot 3.25^2} \approx 4.6$ µm). Synthetic volumes were produced with cylinder radii of 3, 6, 8, 10, 12, and 15 µm, inspired by the radius distributions found in the results of our study (see main **Figs. 2-4**).

Our assessment of the radius quantification accuracy is summarized in **supplemental Fig. 1, C**, presenting the mean bias and its standard deviation for different 3D orientations and radius values. Since the accuracy of the quantified radii strongly depends on the segment’s orientation, more differentiated validation results are presented in **supplemental Fig. 1, D,F**. For each modeled cylinder radius, the mean quantified radii (from respectively 10 cylinders) are plotted with standard deviations for each θ *and* φ angle individually. The validation shows that, under most circumstances, the quantified radii agree very well with the ground truth. Since the algorithm was optimized for structures near the resolution limit, the accuracy is best for small vessels with mean radii $r \lesssim$ 10 µm, where most orientations yield deviations below 1 µm, with an exception for large cylinders at very small angles $\theta \approx$10°. Averaging over all angle orientations, the mean quantification bias and variance are well within the limits of one pixel size, as summarized in **supplemental Fig. 1 C.**

Suppl. references:

1. E. Bullitt, G. Gerig, S. M. Pizer, Weili Lin and S. R. Aylward. Measuring tortuosity of the intracerebral vasculature from MRA images. IEEE Trans Med Imaging. 2003 Sep;22(9):1163-71

2. Lorthois S, Lauwers F, Cassot F Tortuosity and other vessel attributes for arterioles and venules of the human cerebral cortex. Microvasc Res 2014, 91:99–109.

3. C. R. Maurer, Rensheng Qi and V. Raghavan, A linear time algorithm for computing exact Euclidean distance transforms of binary images in arbitrary dimensions, IEEE Transactions on Pattern Analysis and Machine Intelligence, 2003, vol. 25, no. 2, pp. 265-270, Feb.
